# TARGET® Intracranial Aneurysm Coiling Prospective Multicenter Registry: Final Analysis of Peri-Procedural and Long-Term Safety and Efficacy Results

**DOI:** 10.3389/fneur.2019.00737

**Published:** 2019-07-09

**Authors:** Osama O. Zaidat, Alicia C. Castonguay, Ansaar T. Rai, Aamir Badruddin, William J. Mack, Amer K. Alshekhlee, Qaisar A. Shah, Syed I. Hussain, Mouhammed R. Kabbani, Ketan R. Bulsara, Asif M. Taqi, Vallabh Janardhan, Mary S. Patterson, Brittany L. Nordhaus, Lucas Elijovich, Ajit S. Puri

**Affiliations:** ^1^Neuroscience Institute, Bon Secours Mercy Health St. Vincent Hospital, Toledo, OH, United States; ^2^Department of Neurology, University of Toledo, Toledo, OH, United States; ^3^Departments of Radiology, Neurology, and Neurosurgery, West Virginia University, Morgantown, WV, United States; ^4^Neuroscience Department, Presence St. Joseph Medical Center, Joliet, IL, United States; ^5^Department of Neurosurgery, University of Southern California, Los Angeles, CA, United States; ^6^SSM Neuroscience Institutes, DePaul Health, St. Louis, MO, United States; ^7^Abington Memorial Hospital, Abington, PA, United States; ^8^Department of Neurology, Cleveland Clinic-Abu Dhabi, Abu Dhabi, United Arab Emirates; ^9^Gundersen Lutheran Medical Foundation, Inc., La Crosse, WI, United States; ^10^Department of Neurosurgery, University of Connecticut, Farmington, CT, United States; ^11^Desert Regional Medical Center, Palm Springs, CA, United States; ^12^Texas Stroke Institute, Plano, TX, United States; ^13^Vascular Anomalies Center, Le Bonheur Children's Hospital, Memphis, TN, United States; ^14^Department of Radiology, University of Massachusetts, Worcester, MA, United States

**Keywords:** aneurysm, coiling, ruptured aneurysm, occlusion, target coils, target registry, SAH

## Abstract

**Background and Purpose:** To describe the final results of the TARGET Registry, a multicenter, real-world study of patients with intracranial aneurysms treated with new generation TARGET Coils.

**Methods:** The TARGET Registry is a prospective, single-arm study with independent medical event monitoring and core-lab adjudication. Patients with *de novo* intracranial aneurysms were embolized with either TARGET-360° or helical coils in 12 US centers. The primary outcome was aneurysm packing density (PD), which was assessed immediately post-procedure. The secondary outcomes were immediate and long-term aneurysm occlusion rate using the Raymond Scale, and independent functional outcome using the modified Rankin Scale (mRS). A secondary analysis investigated the influence of the use of 100% 360-complex coils on clinical and angiographic outcomes.

**Results:** 148 patients with 157 aneurysms met the inclusion and exclusion criteria. 58 (39.2%) patients with ruptured and 90 (61.8%) with unruptured aneurysms were treated using TARGET 360°, helical Coils, or both. Median age was 58.3 (IQR 48.1–67.4), 73% female, and 71.6% were Caucasian. Median follow-up time was 5.9 (IQR 4.0–6.9) months. The majority were treated with TARGET 360-coils (63.7%), followed by mixed and helical coils only. Peri-procedural morbidity and mortality was seen in 2.7% of patients. A good outcome at discharge (mRS 0–2) was seen in 89.9% of the full cohort, and in 84.5 and 93.3% in the ruptured and unruptured patients, respectively. The median packing density was 28.8% (IQR 20.3–41.1). Long-term complete and near complete occlusion rate was seen in 90.4% of aneurysms and complete obliteration was seen in 66.2% of the aneurysms. No significant difference in clinical and angiographic outcomes were noted between the pure 360-complex coiling vs. mixed 360-complex/Helical coiling strategies. In a multivariate analysis, predictors for long-term aneurysm occlusion were aneurysm location, immediate occlusion grade, and aneurysm size. The long-term independent functional outcome was achieved in 128/135 (94.8%) patients and all-cause mortality was seen in 3/148 (2%) patients.

**Conclusion:** In the multicenter TARGET Registry, two-thirds of aneurysms achieved long-term complete occlusion and 91.0% achieved complete or near complete occlusion with excellent independent functional outcome.

**Clinical Trial Registration:** www.ClinicalTrials.gov, identifier: NCT01748903

## Introduction

Although the use of flow diverters and adjunctive devices has increased in recent years, detachable coils remain the mainstay approach in the endovascular treatment of intracranial aneurysms (1–5). In a study of unruptured intracranial aneurysms, ~60% of anterior circulation aneurysms were 6 mm or less, with 17% prevalence of aneurysms sizes between 1–3 mm and 43% in 4–6 mm in size range (6). These frequently encountered small intracranial aneurysms (≤6 mm) are primarily treated with aneurysm coiling with or without adjunctive devices. Complex coil shapes and 2D helical coils are routinely used to achieve high packing density, complete aneurysm occlusion, and enable the treatment of complex-shaped aneurysms (7). These coil types can be used together in a complementary approach to achieve greater aneurysm occlusion. Previous studies comparing complex-shaped coils to 2D or helical coils are limited. In a matched-pair, case-control study, the use of 3D complex coils as the initial framing coil was associated with better packing density and long-term aneurysm occlusion (7).

The TARGET Intracranial Aneurysm Coiling (TARGET) Registry was a prospective, investigator-initiated, non-randomized, multicenter study with independent clinical event and core-lab adjudication, which aimed to collect real-world data on the use of Target® 360° and Target® helical coils for the embolization of ruptured or unruptured intracranial aneurysms. Here, we present the peri-procedural and long-term safety and occlusion efficacy results from the TARGET Registry.

## Methods

### Study Design

The prospective, multicenter TARGET Registry included ruptured or unruptured saccular intracranial aneurysms, which were treated with Target 360° coils only (360° group) or both Target 360° and Helical coils (Mixed group) (ClinicalTrials.gov Identifier: NCT01748903). A total of 12 clinical sites within the United States participated in the TARGET Registry. All sites received local Institutional Review Board approval for the study. Mercy St. Vincent Medical Center, Toledo, Ohio served as the coordinating center for TARGET Registry.

### Study Population

Patients were eligible for enrollment in the TARGET Registry if the following criteria were met: (1) Age 18 years or older, (2) Previously untreated saccular intracranial aneurysm, unruptured, or ruptured, suitable for embolization with coils, (3) Hunt and Hess Score of 3 or less, (4) Premorbid mRS of 3 or less, (5) Patient or patient's legally authorized representative can provide written informed consent, and (6) The patient is willing to and can comply with study follow-up requirements. Patients were excluded from enrollment if they met any of the following: (1) Less than 18 years of age, (2) Patients with intracranial aneurysms other than the target aneurysm that has undergone treatment ≤ 1 year from enrollment in the study, (3) Dissecting target aneurysm, (4) Patients in whom the target aneurysm will be treated with coils other than Stryker Target® 360° and Target® Helical coils, (5) Target aneurysm is fusiform, (6) Patients in which the target aneurysm cannot be coiled in one procedure (i.e., staged procedure).

### Study Procedures

Standard local institutional trans-arterial coiling techniques and anesthesia approach were used at each site per the participating interventionalist preference. Given that the secondary intention of this study was to assess the results of aneurysms treated with complex shape Target® 360° coils only vs. Mixed 360° and/or Target® 2D Helical coils, investigators were encouraged to use the same coil type for framing, filling, and finishing, if possible.

Use of adjunctive devices was permitted. Long-term follow-up occurred at 3–9 months (per each site's standard of care) and included imaging of the target aneurysm (DSA or MRA) and assessment of clinical outcome using the modified Rankin Scale (mRS). All source data from case report forms were entered into a secure database (REDCAP) and managed/monitored by the central coordinating center.

### Clinical and Imaging Adjudication

An independent medical monitor reviewed all adverse events (AEs) and Serious AEs (SAEs) for the study and adjudicated relatedness to the underlying diseases, procedure, or study device.

For determination of aneurysm occlusion outcome, de-identified vascular angiographic images (DSA and MRA) were sent to the imaging core lab for adjudication of the modified Raymond aneurysm occlusion grade (8, 9) immediately post-procedure and on the long-term imaging follow-up. The progression to complete occlusion or regression to aneurysm recurrence and recanalization was also assessed by the core imaging lab.

### Study Outcomes

The primary study outcomes was aneurysm packing density at immediate post-procedure. Secondary study outcomes included: aneurysm re-access rate (Microcatheter kick-back rate), time of fluoroscopic exposure, overall procedure time, aneurysm recurrence at follow-up, aneurysm re-treatment rate, aneurysm bleed and re-bleed rate, treatment related morbidity and mortality, clinical outcome (mRS) at baseline and follow-up, and peri/post-procedural adverse events related to device and/or procedure.

A secondary analysis was performed comparing technical and clinical endpoints Target 360° and Target Helical coils.

### Statistical Methods

Categorical data are presented for all patients as a percentage (*n* = frequency count) and as a percentage by treatment group (rounded to the nearest whole number). Continuous data are expressed as mean (SD) or median (IQR) if non-normally distributed. Categorical factors were compared between treatment groups with Chi-square tests or Fisher's Exact tests when cell sizes were small. Continuous data were compared between treatment groups with Student *T*-test or Mann Whitney for non-normal data. Logistic regression was used to calculate the odds ratio for poor outcome (mRS 3–6). Factors associated with packing density were analyzed in univariable analysis with Mann Whitney test and Spearman correlation. All *p*-values were two-tailed. A *p*-value < 0.05 was considered statistically significant. Data were analyzed with SAS and JMP v. 13 (Cary, NC).

This study was not designed to detect differences of a certain magnitude between groups with an apriori power calculation. Therefore, the lack of a significant *p*-value (i.e., “no significant difference”) may be due to small sample size.

## Results

From January 2013 to May 2014, 150 patients with 159 ruptured or unruptured aneurysms were prospectively enrolled in the TARGET Registry. Of the 150 patients, 2 were excluded due to a Hunt & Hess score of 4 and the use of non-TARGET coils. Data from 148 patients with 157 aneurysms were included in the per-protocol analysis (100 in the pure 360° coiling group and 57 in the mixed coiling group 360° and 2D helical coiling).

### Overall TARGET Cohort

#### Baseline

Baseline demographics of the overall TARGET Cohort are summarized in Table 1. Median age was 58.3 (48.1–67.4) years, 73% were female, and 71.6% were Caucasian. Of the 148 patients, 39.2% presented with a ruptured intracranial aneurysm (Table 2). The median maximum aneurysm size was 5.6 mm (IQR 4.4–7.9 mm) and neck diameter was 3.2 mm (IQR 2.2–4.2 mm). Most aneurysms were in the anterior circulation (79.1%), with 37.8, 25.0, and 16.9%, in the internal carotid artery, anterior communicating artery, and middle cerebral artery, respectively. Stent-assisted and balloon-assisted coiling was used in 33.8 and 25.7% of patients, respectively. Median packing density in the overall TARGET cohort was 28.8% (IQR 20.3–41%) (Table 3).

**Table 1:** Patient demographics: overall cohort, 360° and mixed coiling groups result.

	Overall *N*: 148 patients (157 aneurysms)^*^	**360^**°**^ coiling *N*: 92 patients (100 aneurysms)**	**Mixed coiling *N*: 56 patients (57 aneurysms)**	***P***
Age, median (IQR)	58.3 (48.1–67.4)	57.9 (48–66.9)	59.1 (48.4–70.4)	0.5
Gender, female	73.0% (108)	75% (69)	69.6% (39)	0.48
Race				0.21
White/Caucasian	71.6% (106)	76.1% (70)	64.3% (36)	
Black/African American	10.8% (16)	7.6% (7)	16.1% (9)	
Hispanic/Latino	13.5% (20)	14.1% (13)	12.5% (7)	
Other	4.1% (6)	2.2% (2)	7.2% (4)	
Medical history				
Headache/migraine	44.6% (66)	48.9% (45)	37.5% (21)	0.19
Previous stroke				
Hemorrhagic	9.5% (14)	7.6% (7)	12.5% (7)	0.53
Ischemic	9.5% (14)	6.5% (6)	14.3% (8)	0.16
Transient ischemic attack	7.4% (11)	6.5% (6)	8.9% (5)	0.86
Family history intracranial aneurysm	17.6% (26)	20.6% (19)	12.5% (7)	0.39
Diabetes	16.9% (25)	17.4% (16)	16.1% (9)	0.72
Hypertension	59.5% (88)	62.0% (57)	55.4% (31)	0.50
Smoking	48.0% (71)	45.7% (42)	51.8% (29)	0.45
Arteriovenous malformation	1.4% (2)	2.2% (2)	0% (0)	0.48
Polycystic kidney disease	0.7% (1)	1.1% (1)	0% (0)	0.29
Hyperlipidemia	37.8% (56)	35.9% (33)	41.1% (23)	0.82
Seizure	6.8% (10)	6.5% (6)	7.1% (4)	0.98
Previously treated aneurysm	7.0% (10)	7% (6)	8% (4)	0.74
Premorbid mRS∧				0.17
0	87.8% (130)	91.3% (84)	82.1% (46)	
1	8.1% (12)	7.6% (7)	8.9% (5)	
2	1.4% (2)	0% (0)	3.6% (2)	
3	2.0% (3)	1.1% (1)	2.6% (2)	

* *One patient excluded for coils other than TARGET were used; and a second patient excluded due to baseline H&H of 4.^∧^mRS, modified Rankin's Scale*.

**Table 2:** Pre-treatment aneurysm evaluation: overall cohort, 360° and mixed coiling groups result.

	**Overall *N*: 148**	**360^**°**^ coiling *n*: 92**	**Mixed coiling *n*: 56**	***P***
Baseline presenting mRS				
0	64.9% (96)	65.2% (60)	64.3% (36)	1.0
1	22.3% (33)	22.8% (21)	21.4% (12)	1.0
2	8.1% (12)	7.6% (7)	8.9% (5)	0.77
3	2.7% (4)	2.2% (2)	3.6% (2)	0.63
4	1.4% (3)	1.1% (1)	1.8% (2)	0.56
5	0.7% (1)	1.1% (1)	0% (0)	1.0
Aneurysm features				
Aneurysm size, mm, median (IQR)	5.6 (4.4–7.9)	5.5 (4.5–7.5)	5.6 (4.2–8.0)	0.52
Aneurysm neck diameter, mm	3.2 (2.2–4.2)	3.0 (2.1–4.4)	3.3 (2.5–4.2)	0.78
Aneurysm volume, mm^3^	56.0 (25.6–150.0)	59.1 (26.5–152.4)	52.8 (21.2–144)	0.24
Multiple aneurysms	6.1% (9)	5.4% (5)	7.1% (4)	0.67
Side, Right	52.7% (78)	46.7% (43)	62.5% (35)	0.09
Left	39.2% (58)	42.4% (39)	33.9% (19)	0.39
Midline	8.1% (12)	10.9% (10)	3.6% (2)	0.13
Location				
ICA	37.8% (56)	40.2% (37)	33.9% (19)	0.60
ACA	25.0% (37)	19.6% (18)	33.9% (19)	0.08
MCA	16.9% (25)	15.2% (14)	19.6% (11)	0.50
Basilar	15.2% (23)	19.6% (18)	8.9% (5)	0.10
Vertebral	4.1% (6)	4.4% (4)	3.6% (2)	1.0
Extradural ICA	0.7% (1)	1.1% (1)	0% (0)	1.0
Bifurcation	58.8% (87)	55.4% (51)	64.3% (36)	0.31
Anterior	79.1% (117)	73.9% (68)	87.5% (49)	0.09
Shape, irregular	58.8% (87)	57.6% (53)	60.7% (34)	0.73
Daughter Sac	5.4% (8)	4.3% (4)	7.1% (4)	0.48
Multilobulated	17.6% (26)	17.4% (16)	17.8% (10)	1.0
Subarachnoid hemorrhage				
Ruptured status	39.2% (58)	40.2% (37)	37.5% (21)	0.74
Hunt & Hess scale				
I	17.2% (10)	24.3% (9)	4.8% (1)	0.09
II	51.7% (30)	51.3% (19)	52.4% (11)	1.0
III	31.0% (18)	24.3% (9)	42.9% (9)	1.0
Technical features				
Stent-assisted coiling	33.8% (50)	33.7% (31)	33.9% (19)	0.98
Stent type				
Cordis	0.7% (1)	1.1% (1)	0% (0)	
Enterprise	5.4% (8)	3.3% (3)	8.9% (5)	
Neuroform	26.4% (39)	28.3% (26)	23.2% (13)	
Pipeline	1.4% (2)	1.1% (1)	1.8% (1)	
Balloon-assisted coiling	25.7% (38)	22.8% (21)	30.3% (17)	0.31
Balloon type				
Hyperform	1.4% (2)	1.1% (1)	1.8% (1)	
Hyperglide	5.4% (8)	2.2% (2)	10.7% (6)	
Scepter XC	2.0% (3)	2.2% (2)	1.8% (1)	
Transform	16.9% (25)	16.3% (15)	17.9% (10)	
Access site, Right	84.5% (125)	89.1% (82)	76.8% (43)	0.06
Left	6.8% (10)	3.3% (3)	12.5% (7)	0.04
Right & left	8.8% (13)	7.6% (7)	10.7% (6)	0.56

**Table 3:** Immediate and long-term post-treatment outcomes: overall cohort, 360° and mixed coiling groups result.

	**Overall *N*: 148**	**360^**°**^ Coiling *n*: 92**	**Mixed coiling *n*: 56**	***P***
**PACKING DENSITY**				
Packing density %	28.8%	27.7%	31.3%	0.30
Median IQR	(20.3%−41.1%)	(21.4%−41.1%)	(19.9%−41.1%)	
**IMMEDIATE OCCLUSION RAYMOND SCALE PER CORE LAB**				
Raymond class I & II	91.7%	90.2%	90.9%	1.0
Raymond class I	66.9%	66.3%	65.5%	1.0
**DISCHARGE CLINICAL FUNCTIONAL ASSESSMENT (MRS)**				
mRS 0–2	89.9% (133)	92.4% (85)	85.7% (48)	0.26
mRS 0–1	84.5% (125)	88.0% (81)	78.6% (44)	0.16
**DISCHARGE DISPOSITION**				
Acute rehabilitation	24.3% (36)	23.9% (22)	25% (14)	0.34
Home	73.0% (108)	72.8% (67)	73.2% (41)	
Extended care facility	2.0% (3)	2.2% (2)	1.8% (1)	
Expired in hospital	0.7% (1)	1.1% (1)	0% (0)	
**PROCEDURAL TECHNICAL AND CLINICAL OUTCOME**				
Symptomatic morbidity and mortality (up to discharge)	2.7% (4)			
Mortality	0.7% (1)			
Any intraoperative perforation	2.7% (4)	2.2% (2)	3.6% (2)	0.63
Symptomatic	1.4% (2)	1.1% (1)	1.8% (1)	
Any thromboembolic event	4.7% (7)	4.3% (4)	5.4% (3)	1.0
Symptomatic	1.4% (2)	1.1% (1)	1.8% (1)	
**LONG-TERM OUTCOMES:**				
Raymond class I & II	90.4%	89.9%	90.9%	0.80
Raymond class I	66.2%	64.6%	70.5%	0.50
**RETREATMENT RATE**				
Retreatment rate	2.4% (3)	2.5% (2)	2.1% (1)	0.9
**OCCLUSION PROGRESSION PER CORE LAB**				
Progressive occlusion	16.8% (21)	18.8 (15)	12.8 (6)	0.46
Stable	68.0% (87)	67.5 (54)	70.2 (33)	0.84
Recanalization	15.2% (19)	13.8 (11)	17.0 (8)	0.62
**LONG-TERM CLINICAL FUNCTIONAL ASSESSMENT (MRS): 135 (83, 52)**				
mRS 0–2	94.8% (128)	97.6% (81)	90.4% (47)	0.52
mRS 0–1	93.3% (126)	96.4% (80)	88.5% (46)	0.49
Adverse event since discharge	3.1% (4)	2.5% (2)	4.3% (2)	0.64

Immediate complete or near complete occlusion (modified Raymond grade I-II) was achieved in 91.7% (144/157) of the treated aneurysms and 8.3% (13/157) had residual dome filling (Raymond grade III). Grade I (complete occlusion) was achieved in 105/157 (66.9%) of the treated aneurysms.

Microcatheter kick-back (requiring re-accessing of the aneurysm) occurred in 20.3%. The symptomatic complication rate within 24 h was 2.7% (4/148). Intra-operative perforation (IOP) occurred in 2.7% of the patients (2/4 were symptomatic). Peri-procedural mortality rate was 0.7% (1/148). Thrombo-embolic events (TEEs) occurred in 4.7% (7/148) of the patients with 2/7 patients or 1.4% being symptomatic.

Good clinical outcome (mRS 0–2) at discharge was seen in 89.9%. Most patients (73%) were discharged home, while one patient (0.7%) expired in the hospital.

#### Full Cohort Long-Term Outcome

Median follow-up was 5.9 months (IQR: 4–6.9 months) in the overall cohort, with 86.0% (135/157) of the aneurysms had available follow-up angiographic occlusion data and 86.6% (136/157) with clinical outcome reported. At follow-up, 90.4% (123/136) of aneurysms had complete or near complete occlusion. Complete occlusion (RR scale I) was achieved in 66.2% (90/136). At follow-up, 84.8% of patients had a better or stable occlusion status. The retreatment rate in the overall cohort was 2.4%. The rate of good clinical outcome was 94.8%. A total of three deaths occurred including a peri-procedural death (2% mortality rate), one related to IOP, one unrelated death, and one unknown cause during the follow up period (Table 3).

### 360 Coiling and Mixed Coiling Groups

A secondary analysis comparing 100% 360° coils (100 aneurysms) to a mixed approach of both complex 360° shape and 2D helical coils (57) demonstrated no significant difference in the baseline variables between the two groups (Table 1).

The median maximum aneurysm size and neck diameter were similar between the two groups, with no signification difference in the aneurysmal location or relation to a branching point (bifurcation vs. sidewall) (Table 2).

Median packing density was 27.7% (IQR 21.2–41.1) in the 360° coiling group vs. 31.3% (IQR 19–41.1) in the Mixed Coiling group (*p* = 0.3) (Table 3). No difference was observed in the immediate post-treatment occlusion rates or rates of good clinical outcome between the two subgroups (Table 3).

At follow-up, no significant difference was shown in the rates of complete to near complete occlusion (Table 3). The complete occlusion rate was similar between the two groups at 64.6 and 70.5% in the 360° and Mixed groups, respectively (*p* = 0.5, Table 3). No statistical difference was seen in the re-treatment rate between the two cohorts (2.5 and 2.1%, *p* = 0.9).

The rate of good clinical outcome (mRS 0–2) at follow-up was 97.6% in the 360° group vs. 90.4% in the Mixed group (Table 3).

### Predictors of Long-Term Aneurysm Occlusion

After adjusting for age, aneurysm size, bifurcation location, packing density, ruptured status, and use of 100% 360° coils: aneurysm size, bifurcation location, and immediate occlusion status remained independent predictors of long-term complete occlusion on digital subtraction angiography (Table 4).

**Table 4:** Multivariable predictors of long-term aneurysm complete occlusion.

**Term**	**Estimate**	**Std. error**	**ChiSquare**	**Prob > ChiSq**	**Lower 95%**	**Upper 95%**
Intercept	2.66	2.36	1.27	0.26	−1.87	7.45
Age	0.00	0.03	0.00	0.95	−0.06	0.06
>65 years of age	0.35	0.41	0.72	0.40	−0.45	1.18
Bifurcation	−0.64	0.25	6.54	0.01^*^	−1.15	−0.16
Ruptured	0.42	0.26	2.64	0.10	−0.08	0.94
Aneurysm size	−0.25	0.13	3.88	0.05^*^	−0.52	−0.01
Immediate occlusion	−1.83	0.45	16.70	<0.0001^*^	−2.80	−1.02
Packing density	−0.03	0.03	1.41	0.23	−0.09	0.02
100% 360 coils	0.26	0.25	1.13	0.29	−0.22	0.76

* *p ≤ 0.05*.

### Illustrative Case

**Figure 1:**
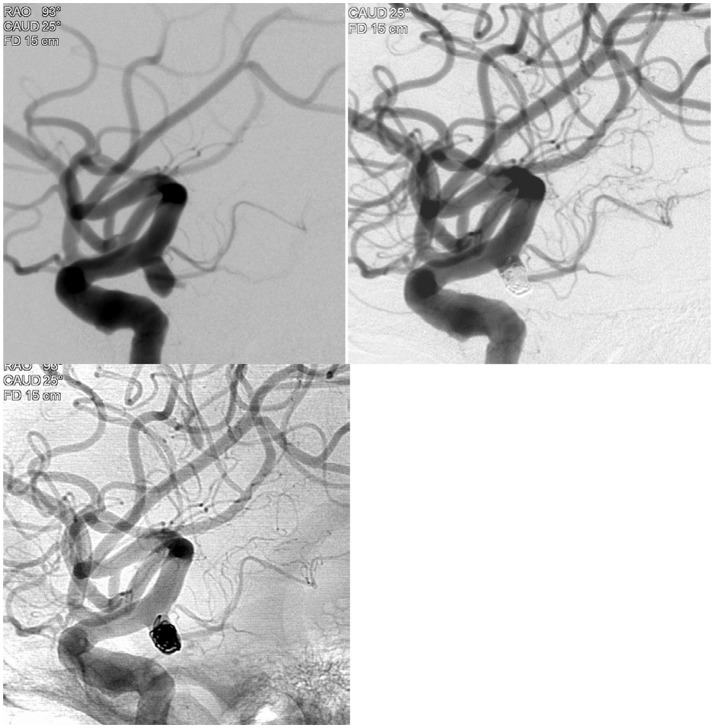
Illustrative case example demonstrating the 100% 360 coiling case in a patient with subarachnoid hemorrhage.

A middle age woman presented with a severe headache and neck stiffness. A plain head CT scan showed diffuse subarachnoid hemorrhage. The CT angiogram demonstrated 5 mm right posterior communicating artery aneurysm. This was treated with 100% 360° Target® coils (360° Ultrasoft coils: 4 mm × 8 cm, 2 × 6, 360° Nano™ coils: 1.5 × 4, 1.5 × 4, 1 × 3, 1 × 2, and 1 × 2), with complete occlusion (Figure 1).

## Discussion

Since the publications of the randomized international (ISAT trial) and US coiling vs. clipping (BRAT) clinical trials (10, 11), coiling technology has continued to evolve with softer and smaller coils allowing for safer and more complete treatment of intracranial aneurysms. The recent literature comparing bare platinum coils vs. bioactive material coated or modified coils to enhance aneurysm occlusion did not yield promising results for bioactive technology (12–16). The Matrix and Platinum Science (MAPS), Cerecyte vs. bare platinum, and hydrogel-coated vs. bare platinum coils randomized clinical trials showed no significant difference in the primary outcomes between bioactive and bare platinum coils (12–14). As bare platinum coils remain the mainstay therapy for intracranial aneurysms, interest has grown in enhancing their mechanical attributes to make them safer, softer, easier to deploy, and therefore, better able to treat smaller aneurysms. The Target coils are the newest iteration of the GDC coils with a softer distal push wire and more supportive proximal wire, with softer and smaller coil diameters.

The TARGET Registry demonstrated complete and near complete occlusion rates in more than 90% of aneurysms at follow-up, with low retreatment rates with TARGET coils. The peri-procedural mortality rate was 0.7% and all-cause long-term mortality rate was 2%. At follow-up, 82.8% of ruptured cases had a mRS ≤ 2, compared to 96% in unruptured cases. These results are comparable to the ISAT and BRAT trial ruptured cohorts, which demonstrated rates of 76.5 and 77%, respectively (10, 11). The slightly better outcome in our ruptured cohort may be due to the differences between the TARGET Registry ruptured population vs. the BRAT and ISAT populations, as well as the smaller sample size in our registry.

The rate of successful occlusion and low retreatment rate are also consistent with other prospective coiling registries using different manufacturer coils. Hirsch et al. in their Trufill™ DCS Orbit platinum coils (Cerenovous, CA) aneurysm registry noted a near complete occlusion rate of 84% with a 5% retreatment rate (17). Similarly, a complete to near complete occlusion rate was seen in 88.4% of the cases in 599 aneurysms treated with Hydrosoft coils® (Microvention CA) with 1.8% rate of morbidity and mortality (18). In the Axium MicroFx Coils (Medtronic, CA) registry, the median follow up was 5 months and revealed 90% of the ruptured aneurysms, and 93.3% of the unruptured aneurysms had Raymond Scale I or II occlusion, with 2% mortality rate occurring exclusively in cases of ruptured aneurysms (19).

### Coil Shape and Outcomes

The main advantages of 3D complex shaped coils are their ability to provide coil mass stability within the aneurysm sac and to allow for coiling of relatively wide-neck aneurysms (20). In a 160 patient study using one or more 3D coils to compare wide neck vs. narrow neck aneurysm groups, the performance of a mixed strategy was similar in both groups regardless of the neck size (20). The 3D framing approach for wide neck aneurysms yielded an angiographic occlusion rate of 68% with morbidity and mortality of 4%, which compared well to the narrow neck aneurysms (20).

However, other authors have reported the value of complex shape coils for improving aneurysm packing density, reducing coils compaction, and potentially increasing the rate of complete aneurysm obliteration with subsequent reduction in the rate of aneurysm re-treatment. Lang et al. demonstrated higher packing density using the 3D coils vs. 2D coils (30 vs. 23%, respectively).

In the TARGET Registry, we evaluated two different strategies, 100% complex shape coils for framing, filling and finishing vs. mixed approach of both complex and helical coils. Our sample size was small and yielded no significant difference between the two approaches in reference to packing density and occlusion rate. These results may also be related to the fact that the coil choice was according to each operator's preference, which may have biased the results. However, we could not identify any significant difference between the two groups when we compared the baseline variables. With advances in complex shape technology, comparing a 100% approach vs. a mixed approach likely requires a larger sample size. Our results suggest that both approaches are acceptable in their safety and efficacy profile.

### Study Limitations

There are several limitations to this study, which include small sample size, the lack of true randomization vs. based on the site and operator preferences, and long-term outcome of less than a year.

## Data Availability

The datasets for this manuscript are not publicly available because the data is part of an investigator-initiated study. Requests to access the datasets should be directed to OZ.

## Ethics Committee

The following Institutional Review Boards approved the protocol for the TARGET Registry: Mercy Health St. Vincent, West Virginia University, Presence St. Joseph Medical Center, University of South California, SSM DePaul Health, Abington Memorial Hospital, Michigan State University, Gundersen Lutheran, Desert Regional Medical Center, Texas Stroke Institute, University of Massachusetts.

## Author Contributions

OZ and AC designed the study, collected and analyzed data, and drafted the manuscript. All other authors participated in data collection and critical review of the manuscript.

### Conflict of Interest Statement

OZ is a consultant for Stryker Neurovascular. WM discloses that he is a consultant for Rebound Therapeutics, Viseon TSP, Medtronic, Penumbra, Stryker, and Stream Biomedical; and an investor in Cerebrotech, Endostream, Viseon, and Rebound. AT discloses that he is a consultant for Stryker and Balt. VJ discloses that he is a Board member, Data Safety Monitoring Board, Penumbra Pivotal Trial, Board member, is an investor in Insera Therapeutics, Inc., Board member, Society of Vascular & Interventional Neurology, Principal Investigator, National Science Foundation (Research Grant). LE reports that he is a consultant for Codman, Stryker, Medtronic, Balt, Microvention, and Scientia Vascular and has Research Support from Siemens. AP discloses that he is a consultant and research grant from Stryker Neurovascular, Research grant from Medtronic and Consultant for Cerenovus. Investor in InNeuroCo and NTI. The remaining authors declare that the research was conducted in the absence of any commercial or financial relationships that could be construed as a potential conflict of interest.
